# Frequency, mutual exclusivity and clinical associations of myositis autoantibodies in a combined European cohort of idiopathic inflammatory myopathy patients

**DOI:** 10.1016/j.jaut.2019.04.001

**Published:** 2019-07

**Authors:** Z. Betteridge, S. Tansley, G. Shaddick, H. Chinoy, R.G. Cooper, R.P. New, J.B. Lilleker, J. Vencovsky, L. Chazarain, K. Danko, M. Nagy-Vincze, L. Bodoki, M. Dastmalchi, L. Ekholm, I.E. Lundberg, N. McHugh, UKMyonet contributors

**Affiliations:** a Department of Pharmacy and Pharmacology, University of Bath, Bath, UK; b Department of Mathematics, University of Exeter, Exeter, UK; c Centre for Musculoskeletal Research, The University of Manchester, Manchester Academic Health Science Centre, Manchester, UK; d National Institute of Health Research Manchester Biomedical Research Centre, Manchester University Foundation Trust.UK; e Salford Royal NHS Foundation Trust, Manchester, UK; f Institute of Ageing and Chronic Disease, University of Liverpool, Liverpool, UK; g Institute of Rheumatology and Department of Rheumatology, 1stMedical Faculty, Charles University, Prague, Czech Republic; h Department of Internal Medicine, University of Debrecen, Debrecen, Hungary; i Division of Rheumatology, Department of Medicine, Solna Karolinska Institutet, And Karolinska University Hospital, Stockholm, Sweden

**Keywords:** Myositis, Dermatomyositis, Polymyositis, Autoantibodies, Autoimmune

## Abstract

**Objectives:**

To determine prevalence and co-existence of myositis specific autoantibodies (MSAs) and myositis associated autoantibodies (MAAs) and associated clinical characteristics in a large cohort of idiopathic inflammatory myopathy (IIM) patients.

**Methods:**

Adult patients with confirmed IIM recruited to the EuroMyositis registry (n = 1637) from four centres were investigated for the presence of MSAs/MAAs by radiolabelled-immunoprecipitation, with confirmation of *anti*-MDA5 and *anti*-NXP2 by ELISA. Clinical associations for each autoantibody were calculated for 1483 patients with a single or no known autoantibody by global linear regression modelling.

**Results:**

MSAs/MAAs were found in 61.5% of patients, with 84.7% of autoantibody positive patients having a sole specificity, and only three cases (0.2%) having more than one MSA. The most frequently detected autoantibody was *anti*-Jo-1 (18.7%), with a further 21 specificities each found in 0.2–7.9% of patients. Autoantibodies to Mi-2, SAE, TIF1, NXP2, MDA5, PMScl and the non-Jo-1 tRNA-synthetases were strongly associated (p < 0.001) with cutaneous involvement. *Anti*-TIF1 and *anti*-Mi-2 positive patients had an increased risk of malignancy (OR 4.67 and 2.50 respectively), and *anti*-SRP patients had a greater likelihood of cardiac involvement (OR 4.15). Interstitial lung disease was strongly associated with the *anti*-tRNA synthetases, *anti*-MDA5, and *anti*-U1RNP/Sm. Overlap disease was strongly associated with *anti*-PMScl, *anti*-Ku, *anti*-U1RNP/Sm and *anti*-Ro60. Absence of MSA/MAA was negatively associated with extra-muscular manifestations.

**Conclusions:**

Myositis autoantibodies are present in the majority of patients with IIM and identify distinct clinical subsets. Furthermore, MSAs are nearly always mutually exclusive endorsing their credentials as valuable disease biomarkers.

## Highlights

• Myositis specific autoantibodies very rarely coexist in the one individual allowing endotypes to be more precisely defined.
• The association of *anti*-TIF1 and cancer-associated myositis is confirmed with a cut-off age of over 58 years.
• In a large combined European myositis cohort associations of *anti*-SRP with carditis and *anti*-Mi-2 with cancer have emerged.
• Myositis associated autoantibodies are strongly associated with having myositis in association with another connective tissue disease.

## 1 Introduction

The idiopathic inflammatory myopathies (IIMs) polymyositis (PM) and dermatomyositis (DM) are heterogeneous conditions characterised by muscle inflammation and weakness, skin rashes and systemic complications including interstitial lung disease (ILD), cardiac involvement and malignancy. Autoimmune mechanisms have a key role in pathogenesis, with the majority of patients developing autoantibodies. These autoantibodies target both nuclear and cytoplasmic components involved in gene transcription, protein translocation and anti-viral responses. Myositis autoantibodies have traditionally been divided into myositis-associated (MAA) and myositis-specific (MSA) autoantibody subsets with the MAAs typically found in myositis patients with overlap features of other connective tissue diseases, and the MSAs predominantly occurring in patients with PM/DM [1,2].

Studies have demonstrated MSA/MAA specificities associate with distinct clinical subsets of patients [[3], [4], [5], [6]] with sero-clinical classifications potentially aiding in prompt diagnosis, as well as helping to predict disease course and response to treatments. However, since myositis is a rare condition, with an incidence of 11 per 1 million person years [7], and some MSAs/MAAs occur in less than one percent of patients, large multicentre cohort studies are required to fully investigate all of the MSA/MAA associations. Herein, we describe the prevalence, mutual exclusivity and clinical associations of myositis autoantibodies in a large European cohort of adult PM and DM patients.

## 2 Materials and methods

### 2.1 Patients and sera

Clinical data and serum or plasma samples from 1637 adult probable or definite PM/DM patients according to the Bohan and Peter criteria [8,9] were available from four large European cohorts recruited to the EuroMyositis registry (United Kingdom (n = 996), Czech Republic (n = 276), Hungary (n = 247) and Sweden (n = 118) (Table 1). Clinical features were recorded using standardised definitions and data collection as described previously [10] and are shown in Supplementary Table 1. Written consent to participate and to provide biological samples was obtained from all subjects according to the Declaration of Helsinki, under the local ethical committee regulations of each participating centre.

**Table 1:** **Demographics of the four European cohorts studied**.

Cohort		United Kingdom	Czech Republic	Hungary	Sweden	Total
**Number**		996	276	247	118	1637
**Gender**	**Female (%)**	67.1	75.5	76.5	69.5	69.6
	**Male (%)**	31.8	20.5	23.5	30.5	29.1
	**Not Known (%)**	1.1	4.0	0.0	0.0	1.3
**Median age at onset (IQR)**		51 (39–61)	61 (50–68)	N/A	N/A	52 (39–63)
**Ethnic Group**	**Caucasian (%)**	81.6	92.8	99.2	98.3	87.4
	**Non-Caucasian (%)**	11.1	0.0	0.0	1.7	6.9
	**Not Known (%)**	7.2	7.2	0.8	0.0	5.7
**Subset**	**DM (%)**	46.3	56.9	31.2	44.9	45.7
	**PM (%)**	53.7	43.1	68.8	55.1	54.3

N/A: Not available, IQR: Inter-quartile range, DM: Dermatomyositis, PM: Polymyositis.

### 2.2 Protein immunoprecipitation (IPP)

IPP using [^35^S]-methionine labelled K562  cell extract was completed as described previously [11,12] to detect autoantibodies against 23 known autoantigens listed in Table 2. Autoantibodies to Ro52 (TRIM-21), 3-hydroxy-3-methylglutaryl-coenzyme A reductase (HMGCR) and cytosolic 5^’^-nucleotidase 1A could not reliably be detected by this method and were therefore omitted from the analysis.

**Table 2:** Autoantibody Frequency and co-existence with another autoantibody in the total cohort of 1673 patients.

Autoantigen specificity^1^	Autoantibody frequency n (%)		Co-Existing Autoantibody																				
			None	MSA														MAA					
				Jo-1	PL7	PL12	EJ	OJ	KS	Zo	Ha	SRP	Mi-2	MDA5	NXP2	TIF1	SAE	PMScl	Ku	Ro60	La	snRNP	Other
**Jo-1**	306 (18.7)		245					1											4	38	13	15	5
**PL7**	22 (1.3)	57 (3.5)	20																	2			
**PL12**	12 (0.7)		10																	1		1	
**EJ**	5 (0.3)		2																1	2			
**OJ**	10 (0.6)		7	(1)															1			2	
**KS**	3 (0.2)		1													1	1						
**Zo**	5 (0.3)		5																				
**Ha**	0 (0.0)		0																				
**SRP**	39 (2.4)		38																			1	
**Mi-2**	88 (5.4)		84																	2		3	
**MDA5**	21 (1.3)		21																				
**NXP2**	38 (2.3)		32																2	3	1		2
**TIF1**	114 (7.0)		105						(1)											3		5	2
**SAE**	42 (2.6)		41						(1)														
**PMScl**	129 (7.9)		119																1	7	2	1	
**Ku**	24 (1.5)		13	(4)			(1)	(1)							(2)			(1)		2		3	
**Ro60**	114 (7.0)		19	(38)	(2)	(1)	(2)						(2)		(3)	(3)		(7)	(2)		30	19	5
**La**	37 (2.3)		1	(13)											(1)			(2)		(30)		2	0
**U1RNP/Sm**	124 (7.6)		65	(15)		(1)		(2)				(1)	(3)			(5)		(1)	(3)	(19)	(2)		18
**Other**	54 (3.3)		28	(5)											(2)	(2)				(5)		18	1

^1^Jo-1: histidyl-tRNA-synthetase, PL7: threonyl-tRNA-synthetase, PL12: alanyl-tRNA-synthetase, EJ: glycyl-tRNA-synthetase, OJ: isoleucyl-tRNA-synthetase, KS: asparaginyl-tRNA-synthetase, Zo: phenylalanyl-tRNA-synthetase, Ha tyrosyl-tRNA synthetase, SRP: signal recognition particle, Mi-2: nucleosome-remodelling deacetyalse complex, MDA5: melanoma differentiation-associated protein 5, NXP2: nuclear matrix protein 2, TIF1: transcriptional intermediary factor 1 alpha and/or gamma subunits, SAE: small ubiquitin-like modifier activating enzyme, PM/Scl: nucleolar macromolecular complex, Ku: DNA-binding nuclear protein complex, Ro60: SSA/Ro60, La: La/SSB, U1RNP/Sm: small nuclear RNA U1RNP and/or Sm subunits. Other includes U3RNP: small nuclear RNA U3 subunit, RNA Pol: RNA polymerase I/II/III, M2 mitochondrial antigen and topoisomerase I. Only three patients (0.18%) had more than one MSA (*anti*-Jo-1 coexistent with *anti*-OJ, *anti*-KS coexistent with *anti*-TIF1 and *anti*-KS coexistent with *anti*-SAE). *Anti*-PmScl is the only MAA that is not detected at all with a MSA.

### 2.3 NXP2 and MDA5 ELISAs

Where IPP resulted in bands at approximately 140 kDa corresponding to NXP2 or MDA5, samples were analysed by ELISA, as described previously [13,14], to confirm the presence or absence of these specificities.

### 2.4 Statistical analysis

Statistical analysis was completed using R [15]. Clinical associations for specific autoantibodies were analysed using 1483 patients that were either positive for a single autoantibody, or were autoantibody negative. Each of the major clinical manifestations was analysed using a generalised linear model using logistic regression. Accordingly, the clinical features of patients within an autoantibody-defined subgroup were compared to the remainder of the cohort allowing adjustment for other autoantibody subgroups. The selection of autoantibodies included in the final models was based on Akaike information criterion by considering all possible subsets [16]. Comparison of IPP autoantibody negative patients against all IPP autoantibody positive patients was performed using 2 × 2 contingency tables and Fisher's exact test. Where applicable, results were expressed as odds ratios (ORs) with 95% confidence intervals (CI).

## 3 Results

### 3.1 Autoantibody prevalence and mutual exclusivity

Key demographics are listed in Table 1. IPP screening resulted in the identification of one or more autoantibodies in 1007 patients (61.5%). Whilst the majority of these cases had a single autoantibody specificity (84.7%), 154 patients (15.3%) had autoantibodies targeting multiple autoantigens; 131 patients had two autoantibodies, 22 patients had three autoantibodies and one patient had four separate autoantibodies.

**Fig. 1:**
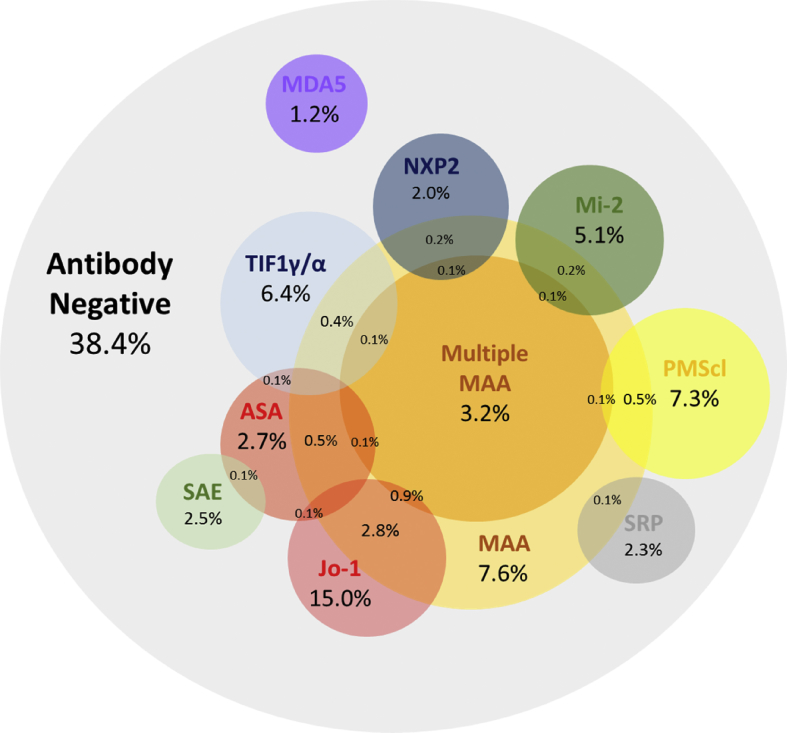
Prevalence and inter-relationship of autoantibodies in myositis. At least one identifiable MSA or MAA is present in 61.5% of myositis patients and myositis specific autoantibodies together with *anti*-PMScl very rarely overlap.

When dividing the autoantibodies into MSAs (*anti*-Jo-1, anti-PL7, anti-PL12, *anti*-EJ, *anti*-OJ, *anti*-KS, *anti*-Zo, anti-Ha, *anti*-SRP, *anti*-Mi-2, *anti*-NXP2, *anti*-MDA5 and *anti*-SAE), and MAAs (*anti*-Ro60, anti-La, *anti*-U1RNP/Sm, *anti*-U3RNP, *anti*-Ku, *anti*-PMScl, *anti*-RNA Pol, AMA and anti-Topo), only three patients had more than one MSA (Table 2). Conversely, the occurrence of an MSA with one or more MAAs was more frequent (98 cases, 6.0% of the total cohort) and multiple MAAs occurred in a further 53 cases (3.2%) (Table 2, Fig. 1).

### 3.2 Myositis autoantibodies identify homogeneous clinical subgroups

The association between autoantibody subsets and clinical features was analysed using linear regression models on data from patients with a single autoantibody. The clinical features associated with different MSAs/MAAs are reported below with strong positive associations defined by p < 0.001. Other data including negative associations, significance levels as p values, ORs and 95% CIs are summarised in Table 3 (MSAs) and Table 4 (MAAs) (and provided in full in supplementary Table 1).

**Table 3:** Clinical Associations of myositis specific autoantibodies.

	Positive Clinical Associations				Negative Clinical Associations			
	Clinical Association	p value	OR	95% CI	Clinical Association	p value	OR	95% CI
***Anti*-Jo-1 n = 245**	ILD	<0.001	13.80	9.84–19.36	V-Sign Rash	= 0.003	0.37	0.19–0.72
	Mechanic's hands	<0.001	8.81	5.59–13.89	Shawl Sign Rash	= 0.047	0.46	0.21–0.99
	Raynaud's	<0.001	2.30	1.62–3.26	Heliotrope Rash	= 0.024	0.63	0.42–0.94
	Arthritis	<0.001	2.04	1.52–2.73				
	Periungual Erythema	= 0.017	1.81	1.11–2.95				
**Non Jo-1 ASA n = 45**	Periungual Erythema	<0.001	64.39	8.44–491.12	Muscle Weakness	= 0.002	0.23	0.10–0.58
	ILD	<0.001	20.58	10.09–41.94				
	Raynaud's	<0.001	7.24	3.07–17.10				
	Mechanic's hands	<0.001	5.88	2.52–13.76				
	Rash (any DM)^1^	= 0.038	1.89	1.03–3.44				
***Anti*-SRP n = 38**	Cardiac Involvement	= 0.004	4.15	1.56–11.04	Gottron's Rash	= 0.017	0.09	0.01–0.65
					Arthritis	= 0.028	0.37	0.15–0.90
***Anti*-Mi-2 n = 84**	Rash (any DM)	<0.001	23.71	10.82–51.99	CTD-Overlap	= 0.030	0.11	0.02–0.81
	Gottron's Rash	<0.001	6.12	3.39–10.15				
	Heliotrope Rash	<0.001	5.64	3.42–9.31				
	Mechanic's hands	<0.001	5.17	2.71–9.87				
	Periungual Erythema	<0.001	4.63	2.63–8.14				
	V-Sign Rash	<0.001	4.13	2.33–7.33				
	Shawl Sign Rash	<0.001	2.87	1.59–5.18				
	Dysphagia	<0.001	3.17	1.86–5.41				
	CAM	= 0.003	2.50	1.35–4.60				
	Cancer (ever)	= 0.013	2.06	1.16–3.63				
***Anti*-MDA5** **N = 21**	Rash (any DM)	<0.001	43.12	5.76–322.62	Raised CK	= 0.038	0.30	0.10–0.93
	Periungual Erythema	<0.001	13.89	3.78–50.97				
	Gottron's Rash	<0.001	11.56	3.84–34.74				
	ILD	<0.001	7.54	3.13–18.19				
	Mechanic's hands	= 0.005	5.81	1.71–19.70				
	Heliotrope Rash	<0.001	5.22	2.08–13.13				
***Anti*-NXP2**	Rash (any DM)	<0.001	7.70	3.29–17.99				
	Heliotrope Rash	<0.001	3.92	1.85–8.28				
	V-Sign Rash	= 0.010	3.50	1.34–9.09				
	Dysphagia	= 0.005	3.30	1.44–7.55				
	Periungual Erythema	= 0.015	3.10	1.24–7.74				
***Anti*-TIF1 n = 105**	Rash (any DM)	<0.001	42.68	17.22–105.83	Raised CK	<0.001	0.26	0.16–0.43
	Gottron's Rash	<0.001	19.49	10.44–36.38	CTD-Overlap	= 0.029	0.27	0.09–0.88
	Heliotrope Rash	<0.001	12.59	7.29–21.77	Muscle Weakness	= 0.001	0.30	0.15–0.62
	Shawl Sign Rash	<0.001	10.24	5.79–18.12	Arthritis	= 0.003	0.46	0.27–0.76
	Periungual Erythema	<0.001	9.56	5.45–16.77	Raynaud's	= 0.034	0.53	0.29–0.95
	V-Sign Rash	<0.001	7.80	4.42–13.77				
	Mechanic's hands	<0.001	6.15	3.44–11.01				
	CAM	<0.001	4.67	2.86–7.63				
	Cancer (ever)	<0.001	4.21	2.69–6.61				
	Dysphagia	<0.001	2.62	1.62–4.23				
***Anti-*SAE n = 41**	Rash (any DM)	<0.001	42.04	10.0–175.15	Arthritis	= 0.025	0.39	0.17–0.89
	Gottron's Rash	<0.001	12.43	5.40–28.59				
	Periungual Erythema	<0.001	15.15	4.93–46.57				
	Shawl Sign Rash	<0.001	9.56	3.74–24.42				
	Heliotrope Rash	<0.001	14.80	3.12–35.79				
	V-Sign Rash	<0.001	5.99	2.38–15.09				

Results are shown on an analysis of 1483 patients with either a single MSA or MAA or no identifiable autoantibody on immunoprecipitation. ^1^Rash (any DM): presence of any one of heliotrope, Gottron's, shawl sign or V sign dermatomyositis rash. ILD: Interstitial Lung Disease, CTD: Connective Tissue Disease, CK: Creatine Kinase OR: Odds Ratio, CI: Confidence Interval, CAM: cancer associated myositis.

**Table 4:** Clinical Associations of myositis associated autoantibodies.

	Positive Clinical Associations				Negative Clinical Associations			
	Clinical Association	p value	OR	95% CI	Clinical Association	p value	OR	95% CI
***Anti*-PMScl n = 119**	Mechanic's hands	<0.001	16.34	9.29–28.76	V-Sign Rash	= 0.049	0.46	0.21–1.00
	CTD-Overlap	<0.001	6.74	4.44–10.22				
	Raynaud's	<0.001	6.44	3.78–10.98				
	ILD	<0.001	6.28	4.12–9.57				
	Dysphagia	<0.001	3.70	2.24–6.11				
	Rash (any DM)^1^	<0.001	2.68	1.82–3.95				
	Periungual Erythema	= 0.002	2.49	1.39–4.45				
	Gottron's Rash	<0.001	2.23	1.47–3.40				
***Anti*-Ku n = 13**	CTD-Overlap	<0.001	9.97	2.63–24.16				
	Arthritis	= 0.009	7.71	1.66–35.90				
	Raynaud's	= 0.013	7.39	1.52–35.92				
	ILD	= 0.007	4.90	1.53–15.72				
***Anti*-Ro60 n = 19**	CTD-Overlap	<0.001	5.42	2.09–14.08				
***Anti*-U1RNP/Sm n = 65**	CTD-Overlap	<0.001	18.17	10.46–31.57	Gottron's Rash	= 0.014	0.31	0.12–0.79
	Raynaud's	<0.001	15.21	5.88–39.35	Heliotrope Rash	= 0.046	0.44	0.19–0.98
	Dysphagia	<0.001	3.37	1.66–6.80				
	ILD	<0.001	2.96	1.67–5.27				

Results are shown on an analysis of 1483 patients with either a single MSA or MAA or no identifiable autoantibody on immunoprecipitation. ^1^Rash (any DM): presence of any one of heliotrope, Gottron's, shawl sign or V sign dermatomyositis rash. ILD: Interstitial Lung Disease, CTD: Connective Tissue Disease, OR: Odds Ratio, CI: Confidence Interval.

#### 3.2.1 Anti-synthetase autoantibodies (ASAs)

The most common autoantibody was *anti*-Jo-1, present in 18.7% of cases (306 patients), with the remaining *anti*-aminoacyl tRNA synthetases (ASAs) (non-Jo-1 ASAs: anti-PL12, anti-PL7, *anti*-EJ, *anti*-OJ, *anti*-KS and *anti*-Zo) collectively found in a further 3.5% of patients (n = 57). No patients had anti-Ha autoantibodies. Due to small numbers the non-Jo-1 ASAs were pooled for analysis. *Anti*-Jo-1 was strongly associated with interstitial lung disease (ILD), mechanic's hands, Raynaud's phenomenon (RP), and arthritis. The non-Jo-1 ASAs were strongly associated with the same features (even more so for ILD) apart from arthritis and strongly associated with periungual erythema.

#### 3.2.2 *Anti*-SRP

*Anti*-SRP autoantibodies were present in 2.4% of patients (39 cases) and were associated with an increased risk of cardiac involvement (p = 0.004, OR 4.15, 95% CI 1.56–11.04).

#### 3.2.3 *Anti*-Mi-2

*Anti*-Mi-2 autoantibodies were present in 5.4% of patients (88 cases), and were strongly associated with an increased risk of DM rash (all subtypes), as well as mechanic's hands, periungual erythema and dysphagia. Patients with *anti*-Mi-2 had an increased risk of both cancer-associated myositis (CAM) (OR 2.5 95% CI 1.35–4.60) or cancer (ever) (OR 2.06 95% CI 1.16–3.63).

#### 3.2.4 *Anti*-MDA5

*Anti*-MDA5 autoantibodies were present in 1.3% of the cohort (21 cases) and were present exclusively in patients with a DM phenotype, and had a strong association with rash (any) as well as specifically Gottron's papules, heliotrope rash and periungual erythema. *Anti*-MDA5 autoantibodies were strongly associated with ILD. *Anti*-MDA5 autoantibodies were the only specificity to be mutually exclusive from any other MSA and MAA, with no *anti*-MDA5 positive patients having co-existing autoantibodies.

#### 3.2.5 *Anti*-NXP2

*Anti*-NXP2 autoantibodies were present in 2.3% of patients (38 cases). *Anti*-NXP2 autoantibodies were strongly associated with rash (any) and specifically heliotrope rash. There were also significant associations with V-sign rash, periungual erythema and dysphagia.

#### 3.2.6 *Anti*-TIF1

*Anti*-TIF1 autoantibodies were present in 7.0% of patients (114 cases), and were strongly associated with all DM subtypes of rash as well as periungual erythema and dysphagia. Additionally, patients with *anti*-TIF1 autoantibodies were at an increased risk of CAM (OR 4.67 95% CI 2.86–7.63) and cancer (ever) (OR 4.21 95% CI 2.69–6.61). The significant association between *anti*-TIF1 autoantibodies and cancer (ever) only existed for patients ≥50 years of age vs patients <50 years of age; OR 3.62 (95% CI 2.09–6.28, p < 0.0001) and OR 1.97 (95% CI 0.56–6.99, p = 0.2940), respectively. The significant association between *anti*-TIF1 autoantibodies and CAM only existed for patients ≥58 years of age vs patients <58 years of age; OR 3.94 (95% CI 1.91–8.16, p < 0.0005) and OR 1.66 (95% CI 0.62–4.40, p = 0.3120), respectively.

### 3.3 7*Anti*-SAE

Autoantibodies to SAE were present in 2.6% of patients (42 cases) and were strongly associated with rash (any), all subtypes of rash and periungual erythema.

### 3.4 MAAs

MAAs were collectively present in 22.5% of patients, with *anti*-PMScl (7.9%), *anti*-Ro60 (7.0%) and *anti*-U1RNP/Sm (7.6%) being the most prevalent, and the remaining specificities (*anti*-Ku, anti-La, *anti*-U3, *anti*-RNA Pol, anti-Topo and AMAs) occurring in less than 2.5% of patients. Since 38.1% of MAA positive patients had dual specificities, only *anti*-PMScl, *anti*-Ku, *anti*-Ro60, *anti*-U1RNP/Sm and *anti*-RNAP-I/III were present in sufficient numbers to allow statistical analysis. All of these autoantibodies were strongly associated with CTD-overlap conditions. *Anti*-U1RNP/Sm and *anti*-PMScl were all strongly associated with RP, ILD and dysphagia. Additionally, *anti*-PMScl autoantibodies were strongly associated with presence of rash (any), mechanic's hands, Gottron's rash and periungual erythema. Other significant associations are shown in Table 4.

### 3.5 No identifiable autoantibody

There was no identifiable MSA or MAA in 627 patients (38.3% of the cohort). Analysis of the clinical associations of this group, in comparison to the collective MSA/MAA positive group, resulted in several negative associations (Table 5) strongly so with mechanics' hands, ILD, periungual erythema, presence of rash (any), Gottron's rash, heliotrope rash, dysphagia, CTD-overlap conditions and RP.

**Table 5:** Clinical associations autoantibody negative patients.

Clinical Feature	Autoantibody Positive (%)	Autoantibody Negative (%)	p value	OR	95% CI
Mechanic's hands	31.4	7.1	<0.001	0.17	0.11–0.25
ILD	39.5	12.2	<0.001	0.21	0.16–0.28
Periungual erythema	49.9	19.9	<0.001	0.25	0.18–0.35
Rash (any DM)^1^	54.4	33.8	<0.001	0.43	0.35–0.53
Gottron's rash	44.4	26.6	<0.001	0.45	0.35–0.58
Dysphagia	45.6	28.4	<0.001	0.47	0.36–0.62
CTD Overlap	18.4	9.7	<0.001	0.48	0.35–0.66
Heliotrope rash	41.8	28.5	<0.001	0.56	0.43–0.71
Raynaud's phenomenon	47.9	33.8	<0.001	0.56	0.43–0.71
Cardiac involvement	12.5	7.5	= 0.018	0.57	0.36–0.91
V-sign rash	37.1	27.4	= 0.008	0.62	0.43–0.88
Shawl sign rash	28.0	19.3	= 0.006	0.64	0.47–0.88

Results are shown on an analysis of the total cohort of 1637 patients comparing patients with at least one identifiable MSA or MAA on immunoprecipitation versus autoantibody negative. ^1^Rash (any DM): presence of any one of heliotrope, Gottron's, shawl sign or V sign dermatomyositis rash. ILD: Interstitial Lung Disease, CTD: Connective Tissue Disease, OR: Odds Ratio, CI: Confidence Interval.

## 4 Discussion

We have shown that autoantibodies specific or associated with myositis as identified by IPP are present in the majority (61.5%) of patients of IIM using four large combined cohorts of patients. Furthermore, MSAs and MAAs identify important clinical phenotypes beyond traditional subgroups of PM/DM. Moreover, the autoantibody negative group was different from the autoantibody positive in having less frequent extra-muscular manifestations. MSAs were found in 42.9% of the total cohort and in those where further clinical details were available were mostly associated with cutaneous features, apart from *anti*-Jo-1 and *anti*-SRP that were associated with arthritis and cardiac involvement respectively. Additionally, *anti*-TIF1 and *anti*-Mi-2 were associated with cancer and *anti*-tRNA synthetases and *anti*-MDA5 with lung disease. MAAs were found in 22.5% of the total cohort and in contrast to MSAs identified patients with CTD/overlap disease, demonstrating a key difference between what is defined as a MSA versus a MAA. The autoantibody negative patients had none of the above characteristics likely reflecting a commonality of myositis in all subgroups given the requirement of fulfilling Bohan and Peter criteria for inclusion in the study.

The MSA/MAAs identified in this mostly Caucasian population were present in expected frequencies with *anti*-Jo-1 (18.7%) the most common [1]. By contrast, Japanese and Chinese cohorts have higher frequencies of *anti*-MDA5 (15–36.6% vs 1.3%) and ASAs (27.6–40.0% vs 22.2%) [17]. Studies of juvenile myositis populations including our own using the identical method of autoantibody detection report higher frequencies of *anti*-TIF1 (18–32% vs 7.0%), *anti*-NXP2 (15–20% vs 2.3%) and *anti*-MDA5 (6% vs 1.3%) [12,18]. Notably ASA are much less frequent in juvenile disease (2–4% vs 22.2%) [12,18]. Why these autoantibodies occur at different frequencies in various cohorts remains unknown, but the data suggest that age, genetics and environmental exposures, may all have key roles in determining autoantibody specificity [5,19].

Another notable finding was that the concurrent presence of more than one MSA in a single patient was extremely uncommon. Whilst 9.4% of our combined cohort had more than one autoantibody, only three cases (0.2%) had more than one MSA. By contrast, MAAs co-existed with other myositis autoantibodies more frequently, although *anti*-PMScl was not present with another MSA. Other studies using IPP to test myositis cohorts have had similar findings with MSAs co-existing in less than 0.2% of cases [20,21], in contrast to data from cohorts screened using other assays where co-existence of MSAs occurs in up to 16.7% of cases [20,22,23], likely reflecting differences in sensitivity and specificities between assays. Nonetheless, the detection of more than one MSA or a MSA with *anti*-PMScl by IPP in an individual patient is rare. A limitation of our study is the absence of results for *anti*-Ro52 autoantibodies, an MAA that is detected frequently in patients with PM/DM, and may confer adverse prognostic importance [24,25].

Autoantibodies to Mi-2, TIF1, MDA5, SAE and NXP2 have traditionally been regarded as ‘DM’ autoantibodies due to their associations with cutaneous features [3] which is consistent with our findings. However, we had insufficient data to investigate reported associations between *anti*-MDA5 and cutaneous ulceration [26] or *anti*-NXP2 and calcinosis [27] which is a limitation of our study. Consistent with previous studies non-Jo-1 ASAs were associated with cutaneous involvement [28] whereas *anti*-Jo-1 was associated with arthritis [29].

The association between IIM and malignancy is well established with a meta-analysis demonstrating a relative risk of 4.66 for DM and 1.75 for PM [30]. The risk is even higher in patients with *anti*-TIF1 with one meta-analysis describing an OR of 27.26 (95% CI: 6.59–112.82) [31]. In agreement, we found *anti*-TIF1 to be strongly associated with malignancy, however at a lower OR in terms of both cancer ever (OR 4.21) and CAM (OR 4.67) that was comparable to an adult American myositis cohort (OR 4.2 for CAM), indicating that patient demographics may have an influence on malignancy risk [32]. We also found a positive association between *anti*-Mi-2 and cancer contrary to previous findings [33]. One other study has reported a positive association between cancer and autoantibodies to the *N*-Terminus of Mi-2 [34], and therefore further investigation ideally including autoantibody reactivity to Mi-2 epitopes is warranted.

A second MSA that has been associated with cancer is *anti*-NXP2. Malignancy was initially reported in 37.5% of Japanese adult *anti*-NXP2 positive patients [35], and was found to be strongly associated in a study on American adult DM patients [32]. However, whilst cancer was more common in our *anti*-NXP2 positive group in comparison to the rest of the cohort (13.5% vs 9.5% for CAM and 17.1% vs 14.2% for cancer-ever), this did not reach statistical significance. These differences may partially be explained by differences in methodology; the prevalence of *anti*-NXP2 in our cohort was similar to other adult myositis cohorts screened by IPP [17,36,37], but was significantly lower than the US cohort assayed by in-vitro IPP [32]. Since multivariate analysis of the US cohort demonstrated the association between *anti*-NXP2 and cancer to be significant only in males, the relatively low number of *anti*-NXP2 positive males in our cohort (n = 11) limited our ability to perform a comparable analysis.

The reported incidence of cardiac involvement in myositis ranges from 6 to 75% depending on patient selection, case definitions and diagnostic testing methods [38]. Whilst initial studies described a correlation between *anti*-SRP and cardiac involvement [1], subsequent investigations have been unable to confirm this finding [39,40]. In our cohort, we found a strong association between *anti*-SRP and cardiac involvement, with patients having a four times increased likelihood for this clinical manifestation.

ILD affects 20–65% of adults with myositis and is associated with a worse prognosis [[41], [42], [43]]. In agreement with previous studies we found the ASAs (Jo-1 and non-Jo-1), PMScl and *anti*-MDA5 to be significantly associated with ILD, with the non-Jo-1 ASA positive patients having an even greater risk of lung involvement than the Jo-1 positive patients [21,44]. *Anti*-MDA5 autoantibodies have been previously associated with ILD in both adult and juvenile cohorts [13,45,46], and rapidly progressive ILD and increased mortality in Eastern Asian patients [13,26,45,47]. We found *anti*-MDA5 positive patients to have a 7.5 fold increased risk of ILD, however lack of details on the severity of ILD prevented us from studying the association with rapidly progressive disease, which is a limitation of our study.

*Anti*-Ku and *anti*-U1RNP autoantibodies have been associated with ILD in SSc and MCTD patients [48,49] and previous studies have described lung involvement in 82% of *anti*-Ku positive and 60% of *anti*-U1RNP positive myositis cases [50,51]. We also found *anti*-Ku and antiU1RNP/Sm to be associated with ILD, although ILD was present in a lower percentage of cases (41.7% for *anti*-Ku and 30.2% for *anti*-U1RNP/Sm). Also the majority of patients with these autoantibodies had CTD-overlap and the ILD association may be with the overlap condition rather than with IIM.

Finally, just under 40% of patients in our cohort had no identifiable autoantibody, although we did not include testing for *anti*-HMGCR, *anti*-Ro52, or *anti*-CN1A that are not reliably detected by our immunoprecipitation assay. However, 74.4% of these patients had autoreactivity to unidentified proteins on immunoprecipitation suggesting the presence of uncharacterised autoantibodies in at least a subset of these cases. Interestingly, whilst these patients were a heterogeneous group, they collectively had a decreased likelihood of overlap disease, cutaneous involvement, ILD and cardiac manifestations, possibly reflecting stronger associations with muscle involvement itself. The lack of sufficient histology to allow a diagnosis of immune-mediated necrotising myopathy is another limitation of our study.

## 5 Conclusion

Myositis patients have been divided traditionally into DM and PM, based on the presence or absence of skin disease. Our results strongly suggest that autoantibodies may offer a better mechanism for identifying clinically relevant and homogenous patient subgroups, borne out by recent studies that have included autoantibodies as part of classification criteria [6,52]. The strong associations of MSA with specific clinical features may help to lead to early identification of patients without classical myopathy features but still at increased risk of potentially life-threatening complications, such as ILD. Further work is warranted to investigate how autoantibody status may influence management decisions and a more personalised approach to therapy.

## Contributors

Czech Republic: heřman mann, olga kryštůfková, martin klein, tereza barochová, kateřina kubínová (institute of rheumatology, prague)

United Kingdom: Janine Lamb, Simon Rothwell (both University of Manchester). UKMYOMET: Yasmeen Ahmed (Llandudno General Hospital), Raymond Armstrong (Southampton General Hospital), Robert Bernstein (Manchester Royal Infirmary), Carol Black (Royal Free Hospital, London), Simon Bowman (University Hospital, Birmingham), Ian Bruce (Manchester Royal Infirmary), Robin Butler (Robert Jones & Agnes Hunt Orthopaedic Hospital, Oswestry), John Carty (Lincoln County Hospital), Chandra Chattopadhyay (Wrightington Hospital), Easwaradhas Chelliah (Wrightington Hospital), Fiona Clarke (James Cook University Hospital, Middlesborough), Peter Dawes (Staffordshire Rheumatology Centre, Stoke on Trent), Christopher Denton (Royal Free London), Joseph Devlin (Pinderfields General Hospital, Wakefield), Christopher Edwards (Southampton General Hospital), Paul Emery (Academic Unit of Musculoskeletal Disease, Leeds), John Fordham (South Cleveland Hospital, Middlesborough), Alexander Fraser (Academic Unit of Musculoskeletal Disease, Leeds), Hill Gaston (Addenbrooke's Hospital, Cambridge), Patrick Gordon (King's College Hospital, London), Bridget Griffiths (Freeman Hospital, Newcastle), Harsha Gunawardena (Frenchay Hospital, Bristol), Frances Hall (Addenbrooke's Hospital, Cambridge), Michael Hanna (University College London Hospitals), Beverley Harrison (North Manchester General Hospital), Elaine Hay (Staffordshire Rheumatology Centre, Stoke on Trent), David Hilton-Jones (Oxford University Hospitals), Lesley Horden (Dewsbury District General Hospital), John Isaacs (Freeman Hospital, Newcastle), David Isenberg (University College London Hospitals), Adrian Jones (Nottingham University Hospital), Sanjeet Kamath (Staffordshire Rheumatology Centre, Stoke on Trent), Thomas Kennedy (Royal Liverpool Hospital), George Kitas (Dudley Group Hospitals Trust, Birmingham), Peter Klimiuk (Royal Oldham Hospital), Sally Knights (Yeovil District Hospital, Somerset), John Lambert (Doncaster Royal Infirmary), Peter Lanyon (Queen's Medical Centre, Nottingham), Ramasharan Laxminarayan (Queen's Hospital, Burton Upon Trent), Bryan Lecky (Walton Neuroscience Centre, Liverpool), Raashid Luqmani (Nuffield Orthopaedic Centre, Oxford), Pedro Machado (University College London Hospitals), Jeffrey Marks (Steeping Hill Hospital, Stockport), Michael Martin (St. James University Hospital, Leeds), Dennis McGonagle (Academic Unit of Musculoskeletal Disease, Leeds), Francis McKenna (Trafford General Hospital, Manchester), John McLaren (Cameron Hospital, Fife), Michael McMahon (Dumfries & Galloway Royal Infirmary, Dumfries), Euan McRorie (Western General Hospital, Edinburgh), Peter Merry (Norfolk & Norwich University Hospital, Norwich), Sarah Miles (Dewsbury & District General Hospital, Dewsbury), James Miller (Royal Victoria Hospital, Newcastle), Anne Nicholls (West Suffolk Hospital, Bury St. Edmunds), Jennifer Nixon (Countess of Chester Hospital, Chester), Voon Ong (Royal Free Hospital, London), Katherine Over (Countess of Chester Hospital, Chester), John Packham (Staffordshire Rheumatology Centre, Stoke on Trent), Nicolo Pipitone (King's College Hospital, London), Michael Plant (South Cleveland Hospital, Middlesborough), Gillian Pountain (Hinchingbrooke Hospital, Huntington), Thomas Pullar (Ninewells Hospital, Dundee), Mark Roberts (Salford Royal Foundation Trust), Paul Sanders (Wythenshawe Hospital, Manchester), David Scott (King's College Hospital, London), David Scott (Norfolk & Norwich University Hospital, Norwich), Michael Shadforth (Staffordshire Rheumatology Centre, Stoke on Trent), Thomas Sheeran (Cannock Chase Hospital, Cannock, Staffordshire), Arul Srinivasan (Broomfield Hospital, Chelmsford), David Swinson (Wrightington Hospital), Lee-Suan Teh (Royal Blackburn Hospital, Blackburn), Michael Webley (Stoke Manderville Hospital, Aylesbury), Brian Williams (University Hospital of Wales, Cardiff), and Jonathan Winer (Queen Elizabeth Hospital, Birmingham).
