# Supplementary material for: Frequency, mutual exclusivity and clinical associations of myositis autoantibodies in a combined European cohort of idiopathic inflammatory myopathy patients
Source: J Autoimmun. 2019 Jul;101:48–55. doi: 10.1016/j.jaut.2019.04.001 (PMC6580360; doi:10.1016/j.jaut.2019.04.001)
Supplement: Multimedia component 2 [file mmc2.docx]

**Supplementary Table 1: Clinical Associations of MSA/MAAs**

| **Clinical feature**  **(present/absent)** | **Jo-1** | **ASA** | **SRP** | **Mi-2** | **MDA5** | **NXP2** | **TIF1** | **SAE** | **PmScl** | **Ku** | **Ro** | **snRNP** | **Ab Negative** |
| --- | --- | --- | --- | --- | --- | --- | --- | --- | --- | --- | --- | --- | --- |
| **Rash (any DM)^1^**  **(668/791)** | **NS** | **p=0.038**  **OR 1.89**  **(1.03-3.44)** | **NS** | **p<0.001**  **OR 23.71**  **(10.82-51.99** | **p<0.001**  **OR 43.12**  **(5.76-322.62)** | **p<0.001**  **OR 7.70**  **(3.29-17.99)** | **p<0.001**  **OR 42.68**  **(17.22-105.83)** | **p<0.001**  **OR 42.04**  **(10.09-175.15)** | **p<0.001**  **OR 2.68**  **(1.82-3.95)** | **NS** | **NS** | **NS** | **p<0.001**  **OR 0.43**  **(0.35-0.53)** |
| **Heliotrope Rash**  **(441/775)** | **p=0.024**  **OR 0.63**  **(0.42-0.94)** | **NS** | **NS** | **p<0.001**  **OR 5.64**  **(3.42-9.31)** | **p<0.001**  **OR 5.22**  **(2.08-13.13)** | **p<0.001**  **OR 3.92**  **(1.85-8.28)** | **p<0.001**  **OR 12.59**  **(7.29-21.77)** | **p<0.001**  **OR 14.80**  **(3.12-35.79)** | **NS** | **NS** | **NS** | **p=0.046**  **OR 0.44**  **(0.19-0.98)** | **p<0.001**  **OR 0.56**  **(0.43-0.71)** |
| **Gottron’s Rash**  **(452/766)** | **NS** | **NS** | **p=0.017**  **OR 0.09**  **(0.01-0.65)** | **p<0.001**  **OR 6.12**  **(3.39-10.15)** | **p<0.001**  **OR 11.56**  **(3.84-34.74)** | **NS** | **p<0.001**  **OR 19.49**  **(10.44-36.38)** | **p<0.001**  **OR 12.43**  **(5.40-28.59)** | **p<0.001**  **OR 2.23**  **(1.47-3.40)** | **NS** | **NS (p=0.076)**  **OR 0.16**  **(0.02-1.21)** | **p=0.014**  **OR 0.31**  **(0.12-0.79)** | **p<0.001**  **OR 0.45**  **(0.35-0.58)** |
| **Shawl Sign Rash**  **(169/533)** | **p=0.047**  **OR 0.46**  **(0.21-0.99)** | **NS** | **NS** | **p<0.001**  **OR 2.87**  **(1.59-5.18)** | **NS** | **NS** | **p<0.001**  **OR 10.24**  **(5.79-18.12)** | **p<0.001**  **OR 9.56**  **(3.74-24.42)** | **NS** | **NS** | **NS** | **NS** | **p=0.006**  **OR 0.64**  **(0.47-0.88)** |
| **V Sign Rash**  **(239/491)** | **p=0.003**  **OR 0.37**  **(0.19-0.72)** | **NS** | **NS** | **p<0.001**  **OR 4.13**  **(2.33-7.33)** | **NS** | **p=0.010**  **OR 3.50**  **(1.34-9.09)** | **p<0.001**  **OR 7.80**  **(4.42-13.77)** | **p<0.001**  **OR 5.99**  **(2.38-15.09)** | **p=0.049**  **OR 0.46**  **(0.21-1.00)** | **NS** | **NS** | **NS** | **p=0.008**  **OR 0.62**  **(0.43-0.88)** |
| **Periungual Erythema**  **(274/472)** | **p=0.017**  **OR 1.81**  **(1.11-2.95)** | **p<0.001**  **OR 64.39**  **(8.44-491.12)** |  | **p<0.001**  **OR 4.63**  **(2.63-8.14)** | **p<0.001**  **OR 13.89**  **(3.78-50.97)** | **p=0.015**  **OR 3.10**  **(1.24-7.74)** | **p<0.001**  **OR 9.56**  **(5.45-16.77)** | **p<0.001**  **OR 15.15**  **(4.93-46.57)** | **p=0.002**  **OR 2.49**  **(1.39-4.45)** | **NS** | **NS** | **NS** | **p<0.001**  **OR 0.25**  **(0.18-0.35)** |
| **ILD^2^**  **(394/1015)** | **p<0.001**  **OR 13.80**  **(9.84-19.36)** | **p<0.001**  **OR 20.58**  **(10.09-41.94)** | **NS** | **NS (p=0.098)**  **OR 0.46**  **(0.18-1.16)** | **p<0.001**  **OR 7.54**  **(3.13-18.19)** | **NS** | **NS** | **NS** | **p<0.001**  **OR 6.28**  **(4.12-9.57)** | **p=0.007**  **OR 4.90**  **(1.53-15.72)** | **NS** | **p<0.001**  **OR 2.96**  **(1.67-5.27)** | **P<0.001**  **OR 0.21**  **(0.16-0.28)** |
| **Cancer (Ever)**  **(180/1061)** | **NS** | **NS** | **NS** | **p=0.013**  **OR 2.06**  **(1.16-3.63)** | **NS** | **NS** | **p<0.001**  **OR 4.21**  **(2.69-6.61)** | **NS** | **NS** | **NS** | **NS** | **NS** | **NS** |
| **Cancer Associated Myositis^3^**  **(139/1287)** | **NS** | **NS** | **NS** | **p=0.003**  **OR 2.50**  **(1.35-4.60)** | **NS** | **NS** | **p<0.001**  **OR 4.67**  **(2.86-7.63)** | **NS** | **NS** | **NS** | **NS** | **NS** | **NS** |
| **Cardiac Involvement^4^**  **(85/754)** | **NS** | **NS** | **p=0.004**  **OR 4.15**  **(1.56-11.04)** | **NS** | **NS** | **NS** | **NS** | **NS** | **NS (p=0.085)**  **OR 1.94**  **(0.91-4.14)** | **NS** | **NS** | **NS** | **p=0.018**  **OR 0.57**  **(0.36-0.91)** |
| **Raynaud’s**  **(288/451)** | **p<0.001**  **OR 2.30**  **(1.62-3.26)** | **p<0.001**  **OR 7.24**  **(3.07-17.10)** | **NS** | **NS** | **NS (p=0.058)**  **OR 2.82**  **(0.96-8.23)** | **NS** | **p=0.034**  **OR 0.53**  **(0.29-0.95)** | **NS (p=0.099)**  **OR 0.40**  **(0.14-1.19)** | **p<0.001**  **OR 6.44**  **(3.78-10.98)** | **p=0.013**  **OR 7.39**  **(1.52-35.92)** | **NS** | **p<0.001**  **OR 15.21**  **(5.88-39.35)** | **p<0.001**  **OR 0.56**  **(0.43-0.71)** |
| **Mechanic’s hands**  **(200/788)** | **p<0.001**  **OR 8.81**  **(5.59-13.89)** | **p<0.001**  **OR 5.88**  **(2.52-13.76)** | **NS** | **p<0.001**  **OR 5.17**  **(2.71-9.87)** | **p=0.005**  **OR 5.81**  **(1.71-19.70)** | **NS** | **p<0.001**  **OR 6.15**  **(3.44-11.01)** | **NS (p=0.064)**  **OR 2.91**  **(0.94-9.00)** | **p<0.001**  **OR 16.34**  **(9.29-28.76)** | **NS** | **NS** | **NS** | **P<0.001**  **OR 0.17**  **(0.11-0.25)** |
| **Arthritis**  **(515/868)** | **p<0.001**  **OR 2.04**  **(1.52-2.73)** | **NS** | **p=0.028**  **OR 0.37**  **(0.15-0.90)** | **NS (p=0.068)**  **OR 0.61**  **(0.36-1.04)** | **NS** | **NS** | **p=0.003**  **OR 0.46**  **(0.27-0.76)** | **P=0.025**  **OR 0.39**  **(0.17-0.89)** | **NS** | **p=0.009**  **OR 7.71**  **(1.66-35.90)** | **NS (p=0.092)**  **OR 0.34**  **(0.10-1.19)** | **NS** | **NS** |
| **CTD Overlap**  **(218/1263)** | **NS** | **NS** | **NS** | **p=0.030**  **OR 0.11**  **(0.02-0.81)** | **NS** | **NS** | **p=0.029**  **OR 0.27**  **(0.09-0.88)** | **NS** | **p<0.001**  **OR 6.74**  **(4.44-10.22)** | **p<0.001**  **OR 9.97**  **(2.63-24.16)** | **p<0.001**  **OR 5.42**  **(2.09-14.08)** | **p<0.001**  **OR 18.17**  **(10.46-31.57)** | **p<0.001**  **OR 0.48**  **(0.35-0.66)** |
| **Raised CK**  **(1247/122)** | **NS** | **NS** | **NS** | **NS (p=0.056)**  **OR 6.88**  **(0.95-49.92)** | **p=0.038**  **OR 0.30**  **(0.10-0.93)** | **NS** | **p<0.001**  **OR 0.26**  **(0.16-0.43)** | **NS** | **NS** | **NS** | **NS** | **NS** | **NS** |
| **Muscle Weakness**  **(1041/63)** | **NS** | **p=0.002**  **OR 0.23**  **(0.10-0.58)** | **NS** | **NS** | **NS** | **NS** | **p=0.001**  **OR 0.30**  **(0.15-0.62)** | **NS** | **NS** | **NS** | **NS** | **NS** | **NS** |
| **Dysphagia**  **(372/615)** | **NS** | **NS (p=0.057)**  **OR 2.18**  **(0.98-4.85)** | **NS** | **p<0.001**  **OR 3.17**  **(1.86-5.41)** | **NS** | **p=0.005**  **OR 3.30**  **(1.44-7.55)** | **p<0.001**  **OR 2.62**  **(1.62-4.23)** | **NS** | **p<0.001**  **OR 3.70**  **(2.24-6.11)** | **NS** | **NS** | **p<0.001**  **OR 3.37**  **(1.66-6.80)** | **p<0.001**  **OR 0.47**  **(0.36-0.62)** |

Data represents the clinical associations of immunoprecipitation positive MSAs and MAAs on 1483 patients with either one or no MSA or MAA. ^1^ Rash (any DM): presence of any one of heliotrope, Gottron’s, shawl sign or V sign dermatomyositis rash. ^2^ILD was defined by chest X-ray or CT, and abnormal pulmonary function tests. ^3^Cancer associated myositis (CAM) was defined as malignancy diagnosed within 3 years of IIM diagnosis. ^4^Cardiac involvement was defined as pericarditis, myocarditis, arrhythmia or sinus tachycardia. Numbers in first column in refer to the documented presence or absence of the listed clinical feature. Cells highlighted in green demonstrate positive associations with cells highlighted in red showing negative associations. ILD: Interstitial Lung Disease, CTD: Connective Tissue Disease, CK: Creatine Kinase OR: Odds Ratio, CI: Confidence Int
